# Network-based transfer of pan-cancer immunotherapy responses to guide breast cancer prognosis

**DOI:** 10.1038/s41540-024-00486-7

**Published:** 2025-01-10

**Authors:** Xiaobao Ding, Lin Zhang, Ming Fan, Lihua Li

**Affiliations:** 1 Institute of Biomedical Engineering and Instrumentation, Hangzhou Dianzi University, Hangzhou, China; 2 Institute of Big Data and Artificial Intelligence in Medicine, School of Electronics and Information Engineering, Taizhou University, Taizhou, China; 3 School of Computer Science and Technology, Hangzhou Dianzi University, Hangzhou, China

**Keywords:** Cancer, Cancer, Biomarkers

## Abstract

Breast cancer prognosis is complicated by tumor heterogeneity. Traditional methods focus on cancer-specific gene signatures, but cross-cancer strategies that provide deeper insights into tumor homogeneity are rarely used. Immunotherapy, particularly immune checkpoint inhibitors, results from variable responses across cancers, offering valuable prognostic insights. We introduced a network-based transfer (NBT) of pan-cancer immunotherapy responses to enhance breast cancer prognosis using node embedding and heat diffusion algorithms, identifying gene signatures netNE and netHD. Our results showed that netHD and netNE outperformed seven established breast cancer signatures in prognostic metrics, with netHD excelling. All nine gene signatures were grouped into three clusters, with netHD and netNE enriching the immune-related interferon-gamma pathway. Stratifying TCGA patients into two groups based on netHD revealed significant immunological differences and variations in 20 of 50 cancer hallmarks, emphasizing immune-related markers. This approach leverages pan-cancer insights to enhance breast cancer prognosis, facilitating insight transfer and improving tumor homogeneity understanding.

Abstract graph of network-based insights translating pan-cancer immunotherapy responses to breast cancer prognosis. This abstract graph illustrates the conceptual framework for transferring immunotherapy response insights from pan-cancer studies to breast cancer prognosis. It highlights the integration of PPI networks to bridge genetic data and clinical phenotypes. The network-based method facilitates the identification of prognostic gene signatures in breast cancer by leveraging immunotherapy response information, providing a novel perspective on tumor homogeneity and its implications for clinical outcomes.
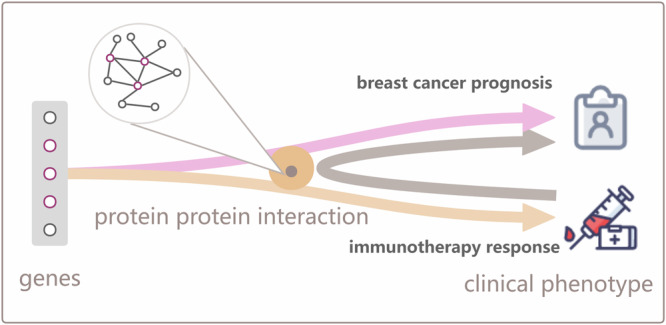

## Introduction

Breast cancer is the most common malignancy among women and remains the leading cause of tumor-related mortality worldwide^1^, despite significant advances in treatment strategies. This disease is notably heterogeneous, with each patient potentially having a different prognosis^2^. Although many great efforts have been made to develop prognostic models for clinical decision-making, accurately predicting the prognosis remains a substantial challenge for patients with breast cancer.

To address this challenge, clinicians and researchers often consider several clinical and pathological features, including tumor size, lymph node status, and histological grade, when assessing prognosis^3,4^. However, these clinicopathological factors are insufficient for accurately predicting the outcomes of patients with breast cancer. Recognizing that tumor heterogeneity originates at the molecular level^5^, molecular examinations of key biomarkers, including estrogen receptor (ER)^6^, progesterone receptor (PR)^7^, HER2^8^, and Ki-67^9^, are routinely performed to enhance the accuracy of prognostic assessments^10^. This approach underscores the critical role these molecular markers play in enabling more precise evaluations of breast cancer prognosis^11^.

In recent years, the advancement of high-throughput molecular profiling technologies has expanded molecular examinations and significantly facilitated the construction of cancer prognosis models^11,12^. Therefore, gene signatures, which consist of multiple genes, are now widely used to predict breast cancer prognosis^13,14^. Researchers can identify these gene signatures through biological experiments or computational methods, both of which are applied within the framework of transcriptomics. For instance, the LM gene signature^15^, which includes 54 genes associated with lung metastasis in breast cancer, was identified from a biological experiment using transcriptomic analysis of cell lines. Compared to biological experiments, computational methods have become prevalent due to their convenience in identifying gene signatures^16^. Numerous gene signatures have been developed, including the PAM50^17^, which builds upon an expanded set of intrinsic genes identified in earlier studies to select those crucial for differentiating among five intrinsic breast cancer subtypes. Endo^18^ conducted univariate Cox regression and ultimately selected eight genes of interest. Similarly, the RS^19^ filters 16 cancer markers from a pool of 250 candidate genes. Additionally, Mamma^20^ and GGI97^21^ leveraged statistical analysis to pinpoint genes with differential expression between two distinct tumor types. Building on these advancements, single-cell RNA sequencing (scRNA-seq) has recently become a key tool for studying tumor heterogeneity at the cellular level^22^. Using scRNA-seq data, researchers have identified the scP.W^23^ gene signature by exploring phenotypic variations associated with epithelial-mesenchymal transition (EMT) in tumor cells. These methods offer a variety of prognostic options for breast cancer, with several having received approval from the Food and Drug Administration for commercial use^24^.

The aforementioned methods identify gene signatures specific to breast cancer prognosis and aim to bridge molecular and clinical phenotypes. These approaches focus primarily on breast cancer and associated genes. However, researchers are increasingly interested in molecular markers suitable for pan-cancer, as these may offer better insights into tumor homogeneity. These markers could also improve breast cancer prognosis. Immunotherapy, especially the use of immune checkpoint inhibitors (ICIs), is effective for pan-cancer treatment and yields distinct outcomes^25^. Patients treated with ICIs can exhibit four distinct response categories—complete response (CR), partial response (PR), stable disease (SD), and progressive disease (PD)—according to the modified RECIST (mRECIST) criteria^26,27^. Immunotherapy responses also reveal distinct prognoses that may advance cancer prognosis research.

Inspired by the success of immunotherapy across various cancers, which has led to distinct responses and prognoses in patients, we hypothesized that certain universal genes and their interactions can consistently predict cancer outcomes. These genes have the potential to guide breast cancer prognosis. However, transferring immunotherapeutic insights to breast cancer prognosis poses significant challenges. To address these challenges, we propose a theoretical model in which the biological network can bridge genes and clinical phenotypes. According to this model, genes and their interactions are pivotal in determining the ultimate clinical outcomes, influencing various clinical phenotypes across different cancer types. Our initial objective within this framework was to identify genes whose expressions anchor immunotherapy responses, which we term anchor genes. After identifying these anchor genes, we can utilize established protein–protein interaction (PPI) databases to gain insights into gene interactions (e.g., STRING^28^) and employ network analysis algorithms to discover additional genes functionally related to these anchor genes. Based on these efforts, we aimed to identify a clinical phenotype-specific gene set and improve the prognostic accuracy for breast cancer patients. The transfer of insights across clinical phenotypes, facilitated by biological networks, offers a new perspective for cancer research, extending beyond breast cancer prognosis.

## Results

### Overview of network-based breast cancer prognosis

**Fig. 1:**
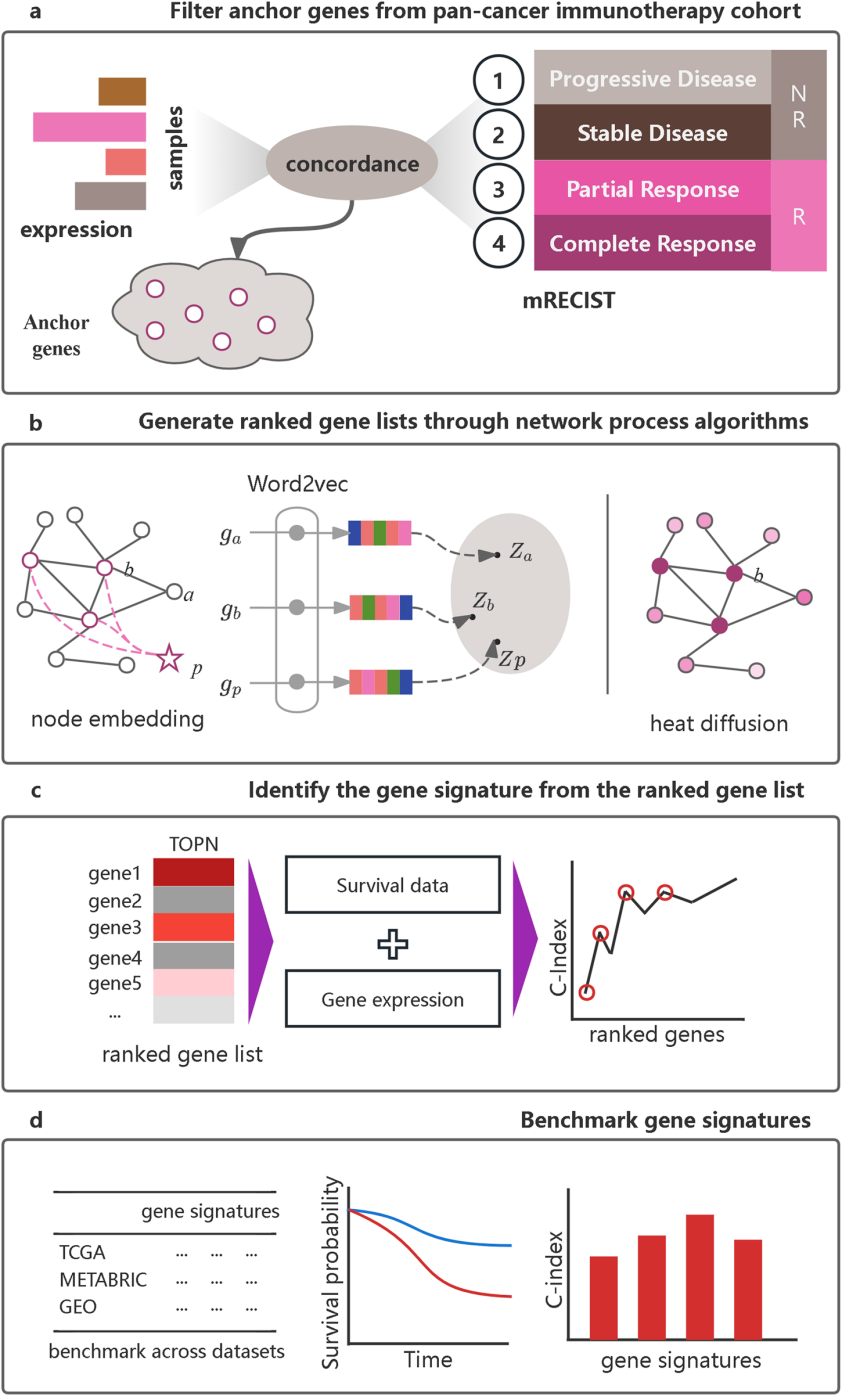
Overview of the NBT workflow. **a** Identification of anchor genes within a pan-cancer immunotherapy cohort. Genes with expression variations that align with immunotherapy responses, as defined by the modified response evaluation criteria in solid tumors (mRECIST), are selected as anchor genes. **b** Generation of ranked gene lists utilizes two distinct strategies: node embedding and heat diffusion. Anchor genes are mapped to the PPIs. In the node embedding strategy, a virtual phenotype node is introduced, with all anchor genes connected to it. The gene list is then ranked based on the similarity between the phenotype node and each gene node. Conversely, the heat diffusion strategy ranks the gene list according to the heat value of each node. **c** Gene signature identification: An improved greedy algorithm is used to identify a gene signature from the ranked gene list, utilizing gene expression and survival data. **d** Benchmark gene signatures: all gene signatures are benchmarked using the concordance index and survival metrics derived from Kaplan–Meier plots across various datasets.

We hypothesized that pan-cancer responses to immunotherapy are determined by specific genes and their interactions, which are applicable to many other clinical phenotypes across different cancer types. Therefore, we propose a network-based transfer (NBT) method utilizing immunotherapy responses to guide breast cancer prognosis. The initial step involved identifying clinical phenotype-specific genes, which we termed anchor genes (Fig. 1a and Supplementary Fig. S1). Specifically, the expression of these genes is directly anchored to immunotherapy responses. Subsequently, a PPI network was abstracted from STRING, with anchor genes mapped to this network (Fig. 1b). Here, we utilized two distinct network analysis algorithms—node embedding and heat diffusion—to generate a ranked gene list for gene signature identification. We then integrated multiple breast cancer cohorts and applied a user-defined greedy strategy to identify gene signatures (Fig. 1c). This process helped us generate distinct gene signatures: netNE, derived from node embedding, and netHD, derived from heat diffusion (Supplementary Table S1). Finally, we benchmarked all gene signatures derived from different methods by comparing them across various breast cancer datasets, including those from the TCGA, METBRIC, and GEO databases (Fig. 1d).

### Prognostic evaluation of pan-cancer immunotherapy cohorts

**Fig. 2:**
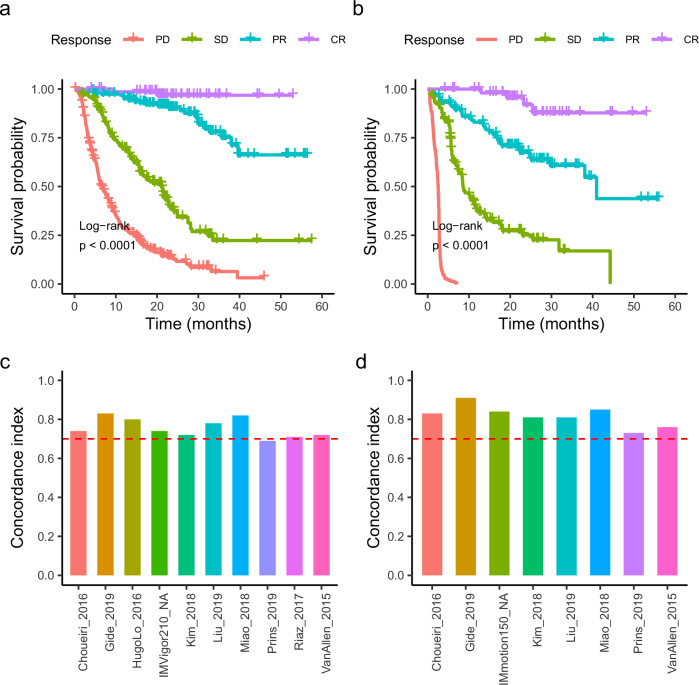
Prognostic evaluation of the pan-cancer immunotherapy cohort. **a** Kaplan–Meier overall survival curves for the cohort, stratified by mRECIST response groups: complete response (CR), partial response (PR), stable disease (SD), and progressive disease (PD). **b** Kaplan–Meier progression-free survival curve for the cohort. **c** The concordance index for each dataset in the cohort, was calculated based on overall survival time. **d** Concordance index for each dataset in the cohort, was calculated based on progression-free survival time.

Immunotherapy represents a groundbreaking treatment approach that is effective for a wide range of cancer types. Despite its broad applicability, it leads to diverse prognostic outcomes among patients. Specifically, patients categorized under CR, PR, SD, and PD showed clear differences in prognosis (Fig. 2a, b). In terms of overall survival or progression-free survival, patients who achieved a CR generally experienced the longest survival, indicating the most favorable prognosis, while those who achieved a PD exhibited the shortest survival, reflecting the least favorable outcomes. Patients with PR and SD fell into the intermediate prognostic category, with PR associated with slightly better outcomes than SD. These findings were consistent across the pan-cancer cohort. To precisely measure the concordance between survival time and immunotherapy responses, the concordance index (C-index) was calculated for each study. All datasets, except for Prins_2019, demonstrated a C-index above 0.7, indicating high concordance, with some studies even surpassing 0.8 (Fig. 2c, d). Immunotherapy elicits responses with distinct prognoses, offering valuable insights into breast cancer prognosis.

### The superior performance of network-based transfer method in risk prediction

To benchmark various methods for breast cancer prognosis, we collected signatures from seven different methods. Our network-based method employed two distinct strategies: netHD, which utilizes heat diffusion, and netNE, which is based on node embedding. The METABRIC dataset, which includes 1420 patients and is the largest cohort, was used to refine both the netHD and netNE. Ultimately, nine signatures were evaluated as risk prediction benchmarks. Both the METABRIC and TCGA datasets provided overall survival (OS) and relapse-free survival (RFS) data, whereas the GPL6098 dataset included only RFS data. We conducted 10-fold cross-validation on each dataset and aggregated the average concordance indices for each gene signature. Additionally, we ranked the gene signatures by their concordance indices and calculated the mean rank for each signature within each dataset (Table 1).

**Table 1:** Performance comparison for cancer prognosis using benchmark methods and network-based approaches (netHD and netNE)

	Endo	GGI97	LM	Mamma	Pam50	RS	scP.W	netNE	netHD
TCGA (OS)	0.58	0.52	0.62	0.50	0.52	0.60	0.57	0.64	0.68
TCGA (RFS)	0.59	0.50	0.57	0.54	0.50	0.57	0.61	0.59	0.61
METABRIC (OS)	0.65	0.62	0.64	0.63	0.64	0.64	0.63	0.65	0.65
METABRIC (RFS)	0.63	0.59	0.60	0.61	0.61	0.61	0.62	0.63	0.63
GEO (GPL6098)	0.65	0.60	0.61	0.63	0.57	0.62	0.63	0.66	0.65
Mean rank	2.4	8.2	5.4	6.4	6.6	4.8	4.4	1.6	1.2

Notably, netHD and netNE demonstrated superior performance, with higher C-indices and lower mean rank values. Specifically, netHD and netNE achieved mean ranks of 1.2 and 1.6, respectively, with netHD performing the best. Following the network-based methods, the Endo method also demonstrated strong performance, particularly with the METABRIC and GEO datasets, where it performed nearly, as well as the network-based methods. However, its performance significantly decreased in the TCGA dataset, with the C-index decreasing to less than 0.6. Additionally, despite containing only eight genes, Endo outperformed larger signatures such as CGI97 (97 genes), Mamma (66 genes), and Pam50 (50 genes).

To further assess the robustness of our approach, we validated the above approach across ten cancer types in TCGA, including SKCM, STAD, and BLCA. Our method was applied in parallel across different cohorts, revealing variability in the C-indices of the prognostic models. Most C-indices exceeded 0.6, with some reaching as high as 0.7 for KIRC and even 0.8 for LGG (Supplementary Table S2). Relative to prognostic models for breast cancer, these results are robust and acceptable, suggesting that transferring immunotherapy responses from a pan-cancer context to inform specific cancer prognoses can effectively broaden our research perspective.

### The network-based transfer method performs well in the risk group

To further evaluate the risk stratification capability of the network-based method, we chose the hazard ratio (HR) as the criterion to benchmark it against seven other methods. For each method, we stratified patients into two groups based on the risk predictions calculated from the respective gene signatures. If a patient’s risk score was greater than the median value, the patient was assigned to the high-risk group; otherwise, the patient was assigned to the low-risk group. HRs were calculated for all predictions generated by each method (Table 2). We observed that the two versions of the network-based method (netHD and netNE) perform well but not outstandingly. The Endo method performed the best, followed by the scP.W. Although the network-based methods did not achieve the best performance, they ranked reasonably well; specifically, they ranked 4th to 5th out of the nine methods according to the mean rank, which is considered acceptable.

**Table 2:** HR evaluation of the network-based method with seven benchmark methods

	Endo	GGI97	LM	Mamma	Pam50	RS	scP.W	netHD	netNE
TCGA (OS)	1.87	0.89	0.95	0.89	0.85	1.06	2.47	1.13	1.30
TCGA (RFS)	2.72	0.90	1.05	1.11	0.88	1.75	2.27	1.31	1.31
METABRIC (OS)	2.16	1.31	1.47	1.54	1.45	2.00	1.96	1.71	1.78
METABRIC (RFS)	2.17	1.24	1.58	1.57	1.54	2.02	2.11	1.74	1.84
GEO (GPL6098)	1.95	1.00	1.03	1.17	1.04	1.49	1.83	1.32	1.70
Mean rank	1.2	8.4	6.8	6.4	8.2	3.4	2	4.6	3.6

### Evaluation using an independent test

In the aforementioned benchmark analysis, we considered both the C-index and HR to evaluate the comprehensive performance of various methods. Among these, netHD, which utilizes a heat diffusion strategy, performed better. We further evaluated its performance with other methods using an independent test. The entire METBRIC dataset was used as the training dataset. All nine gene signatures were used to train prognostic models, which were then evaluated on the full TCGA dataset and across various cancer subtypes using the C-index. Subsequently, breast cancer patients from the TCGA dataset were divided into two groups according to their risk predictions. We then assessed the survival outcomes of these two groups.

**Fig. 3:**
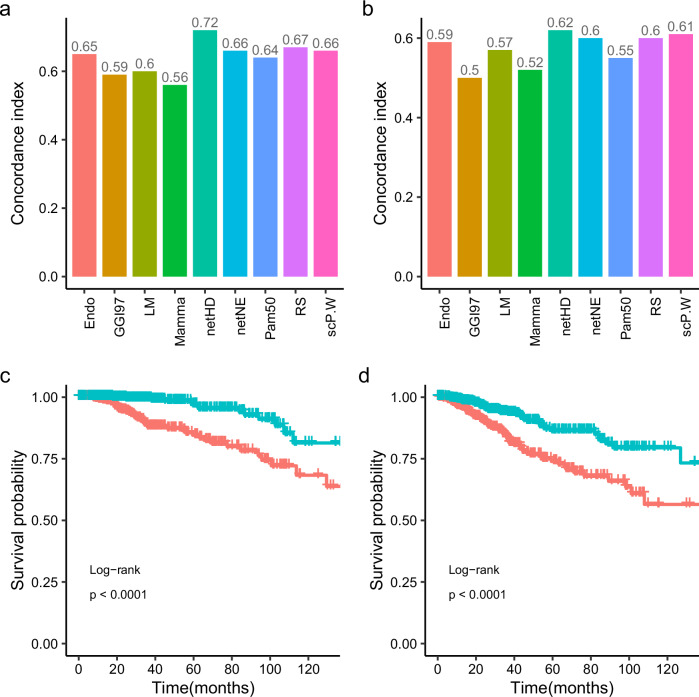
Independent test for nine gene signatures. **a** Concordance index in TCGA (OS): prognosis models developed with nine distinct gene signatures, initially trained on the METABRIC dataset, were evaluated on the TCGA dataset to assess their concordance indices for overall survival (OS). **b** Concordance index in TCGA (RFS): similar to (**a**), models were tested for relapse-free survival (RFS) in TCGA. **c** Survival analysis in TCGA (OS): Kaplan–Meier curves for overall survival, categorizing patients into prognostic risk groups based on netHD model predictions. **d** Survival analysis in TCGA (RFS): similar to (**c**), Kaplan–Meier curves for relapse-free survival.

The netHD achieved the best performance on both the TCGA (OS) and TCGA (RFS) datasets (Fig. 3a, b). Notably, for the TCGA (OS), the netHD method significantly outperformed the other methods, achieving a C-index of 0.72. Following closely, RS and scP.W also performed well, with concordance indices of 0.67 and 0.66, respectively, on the TCGA (OS), and 0.6 and 0.61 on the TCGA(RFS). Based on the risk scores, patients were categorized into high and low-risk groups, each showing distinct survival outcomes with a *p*-value of less than 0.0001(Fig. 3c, d).

Considering the heterogeneity of breast cancer, the test dataset was further categorized by ER, PR, and HER2 status (positive or negative) into four subtypes: ER+, PR+, HER2+, and triple-negative breast cancer (TNBC). We conducted evaluations on these subtypes, where netHD and netNE performed well compared to other models, with the netNE gene signature showing the best performance (Table 3).

**Table 3:** Independent testing on the TCGA breast cancer subtype datasets

	Endo	GGI97	LM	Mamma	Pam50	RS	scP.W	netHD	netNE
ER+ (OS)	0.6	0.55	0.59	0.55	0.65	0.65	0.62	0.62	0.67
ER+ (RFS)	0.57	0.52	0.59	0.51	0.53	0.6	0.61	0.6	0.64
PR+ (OS)	0.57	0.56	0.63	0.62	0.69	0.64	0.67	0.62	0.66
PR + (RFS)	0.58	0.56	0.59	0.53	0.54	0.57	0.64	0.62	0.66
HER2+ (OS)	0.85	0.65	0.81	0.65	0.71	0.82	0.73	0.74	0.73
HER2+ (RFS)	0.67	0.54	0.74	0.53	0.57	0.68	0.7	0.67	0.71
TNBC (OS)	0.57	0.7	0.58	0.59	0.61	0.59	0.55	0.71	0.55
TNBC (RFS)	0.52	0.66	0.52	0.53	0.52	0.61	0.56	0.64	0.53
Mean rank	5.8	6.5	4.9	7.5	5.5	3.6	3.9	3.6	3.4

### Functional co-occurrence analysis of gene signatures

Although gene signatures identified by different methods often vary in quantity, they typically represent biological functions. Among the nine gene signatures, we aimed to further quantify their functional co-occurrence. To this end, we utilized the METABRIC and TCGA datasets to evaluate the functional co-occurrence of these gene signatures. Each sample from these two cohorts was assigned an enrichment score for each gene signature. Subsequently, we evaluated the functional correlations between gene signatures using these scores.

**Fig. 4:**
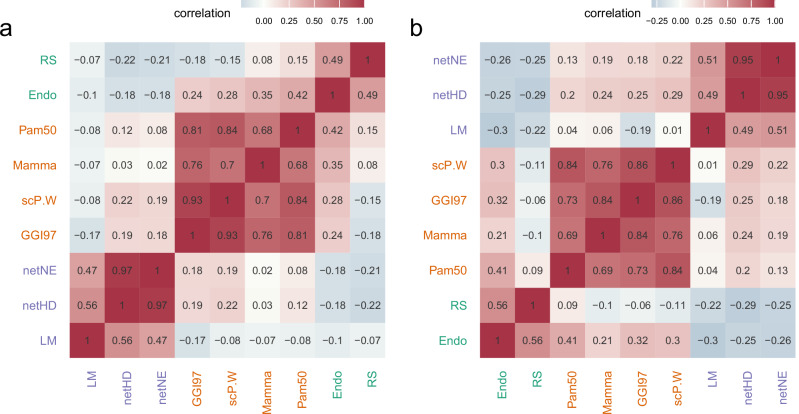
Functional co-occurrence analysis. **a** Functional co-occurrence of nine gene signatures within the TCGA cohort, with color intensity indicating the degree of co-occurrence; darker colors denote greater degrees of co-occurrence. The different text colors denote different clusters. **b** Similar to (**a**), the functional co-occurrence of the same nine gene signatures within the METABRIC cohort.

The results indicate that these gene signatures can be grouped into three clusters based on their functional correlations. Notably, netHD and netNE showed the highest similarity, with scores of 0.97 in the TCGA dataset (Fig. 4a) and 0.95 in the METABRIC dataset (Fig. 4b). However, these two signatures exhibit low similarity with others, except for the LM gene signature, which has a moderate similarity score of approximately 0.5 with both netHD and netNE. CGI97, scP.W, Mamma, and PAM50 formed a distinct cluster, demonstrating high similarity and significant functional co-occurrence. Furthermore, all these gene signatures were enriched in the biological process of cell cycle checkpoints (Supplementary Table S3). Similarly, Endo and RS showed a notable similarity, each achieving a score of 0.5 in both the TCGA and METABRIC datasets. This analysis of functional co-occurrence suggested that multiple functions likely contribute to breast cancer prognosis.

### Gene signature function and tumor microenvironment analysis

Multiple functions derived from various gene signatures may collectively influence breast cancer prognosis. The netHD and netNE exhibit high similarity in terms of functional co-occurrence, with netHD achieving the best performance according to the aforementioned benchmarks. We further analyzed the functions of netHD and examined the tumor microenvironment to gain deeper insights into breast cancer prognosis.

**Fig. 5:**
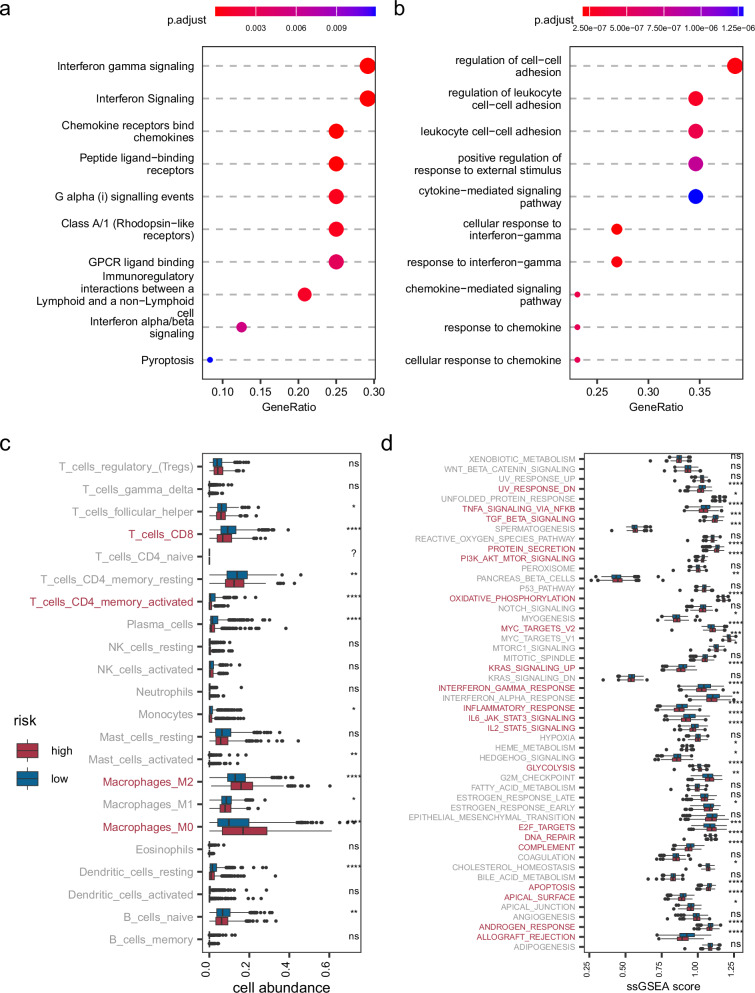
Functional enrichment and tumor microenvironment (TME) analysis. **a** Gene set enrichment analysis for netHD using the Reactome database. **b** Similar to (**a**), enrichment analysis using the Gene Ontology (GO) database. **c** Cellular abundance in the TME between high and low prognosis risk groups. **d** Cancer hallmark enrichment analysis for high and low prognostic risk groups.

To thoroughly assess the function of netHD, we utilized two distinct databases for gene set enrichment analysis: the human-curated REACTOME^29^ database and the computational database GO^30^. Our findings indicate that netHD predominantly captures immune-related annotation terms. Specifically, gene set enrichment analysis using REACTOME revealed that the top three enriched terms were interferon gamma signaling, interferon signaling, and chemokine receptor binding to chemokines (Fig. 5a). Similarly, analysis conducted with the GO database highlighted that the terms associated with cell–cell interactions, particularly regulation of cell-cell adhesion, regulation of leukocyte cell-cell adhesion, and leukocyte cell-cell adhesion, ranked among the top three (Fig. 5b). Furthermore, terms associated with interferon-gamma were among the top ten enriched terms in both the Reactome and GO databases.

The netHD gene signature, which represents immune-related functions, plays a crucial role in breast cancer prognosis. We delved deeper into the tumor microenvironment (TME) across various prognostic risk groups by examining the abundance of immune cells and the activity of cancer hallmarks. Specifically, CIBERSORT^31,32^ was used for cell deconvolution, and the 50 gene signatures^33^ associated with cancer hallmarks were used to evaluate cancer activity. Patients in the TCGA cohort were categorized into high-risk and low-risk groups based on their risk scores. Significantly, by setting the *p*-value cutoff below 0.0001, we observed distinct immunological profiles between the high-risk and low-risk breast cancer groups. The high-risk group demonstrated reduced levels of CD4 + T cells and CD8 + T cells but showed an increased abundance of M2 and M0 macrophages (Fig. 5c). Conversely, the low-risk group exhibited the opposite trend, underscoring the critical influence of these cells on breast cancer prognosis. Furthermore, with the same stringent *p*-value cutoff, notable differences in cancer hallmarks were evident between the two groups, with 20 out of 50 hallmarks showing significant variation (Fig. 5d). Among these, the INTERFERON_GAMMA_RESPONSE hallmark aligned consistently with gene set enrichment terms related to interferon-gamma. Additionally, the hallmarks IL2_STAT5_SIGNALING and IL6_JAK_STAT3_SIGNALING were identified as cytokine-mediated processes integral to the immune response, highlighting their potential roles in modulating cancer risk profiles.

## Discussion

To accurately predict breast cancer prognosis, we introduced a novel approach by transferring observations from pan-cancer immunotherapy responses to breast cancer prognosis. This approach employs a network-based method that utilizes both heat diffusion and node embedding to identify gene signatures. Compared with other established methods, these gene signatures have demonstrated promising performance in various evaluations.

Current methods for identifying gene signatures in breast cancer prognosis are cancer-specific and often miss insights into tumor homogeneity across multiple cancer types, an area of growing research interest. Our approach extends this focus to include pan-cancer immunotherapy responses. Existing methods, such as LM^15^for distant metastasis, GGI97^21^ for a histologic grade, and Endo^18^ for survival, primarily concentrate on the survival outcomes and malignant clinical phenotypes of breast cancer. Unlike methods that directly use clinical phenotypes, scP.W^23^ focuses on the EMT phenotype of breast cancer cells, highlighting EMT as a key factor in tumor homogeneity and prognosis. However, this method is specifically applied to identify gene signatures within a breast cancer-specific single-cell atlas. Transferring clinical phenotypes across cancer types provides an alternative approach to breast cancer prognosis, reflecting tumor homogeneity through interferon gamma-related processes. interferon-gamma (IFNγ) plays a critical role in the anti-tumor immune response during immunotherapy. Immune checkpoint blockade has been shown to upregulate IFNγ, which in turn facilitates the clearance of tumor cells across various cancers^34^. Moreover, IFNγ has been identified as a prognostic marker in melanoma^35^ and the majority of breast cancer cases^36^. This shared feature between immunotherapy and prognosis underscores the consistent role of IFNγ in influencing clinical outcomes.

The biological network serves as the bridge between genes and clinical phenotypes across various cancer types. Tumor heterogeneity often arises from the gap between genes and clinical phenotypes^37^. A biological network was introduced, revealing that only a minority of established clinical phenotype-specific genes can be extended to gain deeper insights^38^. Additionally, these insights can be transferred across cancer types to guide other clinical predictions. Based on this, the tumor homogeneity characteristics hidden in immunotherapy responses can be transferred to breast cancer prognosis. Similarly, the deep neural network framework has successfully made predictions from pixels to objects^39,40^, effectively bridging the gap between lower pixel features and higher object features. Unlike deep neural networks, whose parameters require large amounts of data to learn, biological networks are inherently meaningful and incorporate a wealth of prior knowledge. This makes it feasible to robustly identify gene signatures for breast cancer prognosis.

Many biological processes can influence breast cancer prognosis, with immune-related biological processes being a significant factor in determining clinical phenotypes across cancer types. We collected nine gene signatures representing different functional co-occurrence patterns. Some of these are enriched in the cell cycle checkpoints biological pathway, indicating that the cancer cell proliferation phenotype influences breast cancer prognosis^41^. The gene signatures netHD and netNE, which we proposed, do not co-occur with others. They are enriched in interferon-gamma and chemokine biological processes. Notably, the shared genes within netHD and netNE include key markers associated with CD8 + T cells, underscoring the critical role of CD8 + T cells in tumor immunity. These genes not only indicate CD8 + T cell infiltration (e.g., CD8A, CXCL9^42^, and CXCL10^43^) but also reflect CD8 + T cell activity, with positive regulators such as IFNG^44^ and IRF1^45^. Interestingly, LAG3 appears as an inhibitory marker^46^. Collectively, these genes provide a comprehensive view of the CD8 + T cell landscape within the tumor microenvironment across cancer types. CD8 + T cells are the most powerful effectors in the anticancer immune response and form the backbone of current successful cancer immunotherapies^47^. In terms of cancer prognosis, CD8 + T cell infiltration has also been identified as an independent prognostic marker for glioma^48^, melanoma^49^, kidney renal cell carcinoma^50^, and the majority of breast cancer cases^51^. This finding further suggests that immune-related gene signatures provide valuable insights into breast cancer prognosis. Nevertheless, HER2+ and TNBC, as subtypes of breast cancer, do not fully support this conclusion, as they exhibit distinct immune-related pathway activities compared to other subtypes. This suggests the need for subtype-specific approaches when using immune-related gene signatures for prognosis prediction.

In summary, we adopted a new perspective on breast cancer prognosis by leveraging responses from pan-cancer immunotherapy, utilizing a network-based method to transfer clinical phenotypes across different cancer types. The gene signature we identified not only performed well in cancer prognosis but also uniquely showed enrichment in immune-related pathways. Looking ahead, a predictive model for cancer immunotherapy could be developed by applying single-sample gene set enrichment analysis (ssGSEA)^52,53^, machine learning^54,55^, or neural networks^56,57^ based on our gene signatures. These approaches could also be adapted for predicting breast cancer immunotherapy responses. Additionally, the gene signatures we identified could be used to develop a prognosis model specifically tailored for breast cancer subtypes.

## Methods

### Pan-cancer immunotherapy cohort

Pan-cancer immunotherapy responses play a crucial role in filtering anchor genes and transferring clinical phenotypes. We systematically collected a total of 948 samples, including 11 cohorts from immune checkpoint therapy studies. Among these patients, 275 responded to immunotherapy, and 673 were non-responders. These cohorts included five melanoma cohorts (SKCM): Liu2019^58^, Riaz2017^59^, Gide2019^60^, Van Allen2015^61^, and HugoLo2016^62^; three renal cell carcinoma cohorts (KIRC): IMmotion150^63^, Miao2018^64^, and Choueiri2016^65^; one gastric cancer cohort (STAD)^66^; one bladder cancer cohort (BLCA): IMVigor210^67^; and one glioma cohort (GBM): Prins2019^68^. The entire set of immunotherapy responses, was evaluated according to mRECIST (Supplementary Table S4). To facilitate subsequent analyses, we converted all transcriptome data into TPM format.

### Breast cancer cohorts

To train and test the identified prognostic gene signatures, we aggregated breast cancer cohort data from various sources and molecular profiling techniques. Specifically, we collected breast cancer data from 987 patients from the TCGA cohort, 1420 patients from the METABRIC, and 216 patients from the GEO data platform GPL6098, with accession number GSE22219^69^. The TCGA and METABRIC cohorts provided data on overall survival (OS) and relapse-free survival (RFS), while the GPL6098 cohort provided only RFS data (Supplementary Table S5). Finally, to address batch effects across these datasets, we first applied a log2(*x* + 1) transformation to stabilize variance and normalize the data, followed by batch effect correction using Combat^70^.

### Identification of anchor genes

The anchor genes are phenotype-specific genes, with expression anchored to variations in clinical phenotypes across samples. These genes were identified from the pan-cancer immunotherapy cohort. According to mRECIST, immunotherapy responses are categorized into four groups: complete response (CR), partial response (PR), stable disease (SD), and progressive disease (PD).

To identify anchor genes, we quantified the immunotherapy responses using a digital scale (CR = 4, PR = 3, SD = 2, and PD = 1) based on the degree of response. We designed a user-defined consistent index to measure the consistency between gene expression and phenotypic variation across samples.

1 $${{\bf{C}}}_{{\bf{g}}}=\frac{\sum [({{\bf{g}}}_{{{\bf{p}}}_{{\bf{i}}}}-{{\bf{g}}}_{{{\bf{p}}}_{{\bf{j}}}})\cdot ({{\bf{r}}}_{{{\bf{p}}}_{{\bf{i}}}}-{{\bf{r}}}_{{{\bf{p}}}_{{\bf{j}}}}) > {\bf{0}}]}{{{\bf{C}}}_{{\bf{N}}}^{{\bf{2}}}}$$

The formula (1) defines ${{\boldsymbol{C}}}_{{\boldsymbol{g}}}$ the consistency value for a specific gene ${\boldsymbol{g}}$ within patients from the pan-cancer immunotherapy cohort. For each pair of patients ${{\boldsymbol{p}}}_{{\boldsymbol{i}}}$ and ${{\boldsymbol{p}}}_{{\boldsymbol{j}}}$, the product of the difference in gene ${\boldsymbol{g}}$'s expression level $({{\boldsymbol{g}}}_{{{\boldsymbol{p}}}_{{\boldsymbol{i}}}}-{{\boldsymbol{g}}}_{{{\boldsymbol{p}}}_{{\boldsymbol{j}}}})$ and the difference in immunotherapy responses grade $({{\boldsymbol{r}}}_{{{\boldsymbol{p}}}_{{\boldsymbol{i}}}}{\boldsymbol{-}}{{\boldsymbol{r}}}_{{{\boldsymbol{p}}}_{{\boldsymbol{j}}}})$ is calculated. We count only those pairs where the product of differences is positive, as indicated by the specified indicator function $[\cdot\,>\,0]$. The denominator${{\boldsymbol{C}}}_{{\boldsymbol{N}}}^{{\boldsymbol{2}}}$, representing the total count of all possible unique patient pairs within the immunotherapy cohort, effectively normalized the sum. Ultimately, genes that exhibited a ${{\boldsymbol{C}}}_{{\boldsymbol{g}}}$ exceeding 0.6 were identified as anchor genes. In most prognostic models, a *C*-index of around 0.6 or higher is generally considered acceptable for predictive performance, which guided our decision to adopt this threshold directly^71^ (see Supplementary Table S6 for a list of all anchor genes).

### Node embedding for PPI nodes

To identify gene signatures, a ranked list of genes related to clinical phenotypes is crucial for gene filtering. We mapped the anchor genes onto PPIs and added a virtual node connected to all anchor genes. The PPIs were extracted from STRING with a threshold score of 700. Each node in this new network was vectorized using the DeepWalk algorithm. Each node was sampled 100 times with a sampling length of six genes. We employed the Word2Vec method from gensim, setting a vector size of 256 for rich semantic detail and a window size of 4 to focus on close contextual relationships. The Skip-gram model (sg = 1) with negative sampling (negative = 10) was used to enhance robustness, while an initial learning rate of 0.03, decreasing to 0.0007, ensured stable convergence. The network was trained for 50 epochs to obtain embedding vectors for each node. These embedding vectors facilitate the generation of a gene ranking list for clinical phenotypes.

### Heat diffusion in PPI networks

Unlike the node embedding strategy, heat diffusion offers an alternative approach for obtaining a ranked gene list based on the heat value of each node. It is an important and widely used algorithm in systems biology, with applications in protein function prediction, disease gene prioritization, and patient stratification. Due to its algorithmic advantages in terms of memory usage, the heat diffusion model is much faster to compute, which is why we have chosen it for our analysis. The calculation is as follows (2):

2 $${d}={h}* {exp} (-{Lt})$$

where ${\boldsymbol{h}}$ represents a vector of the original query, and ${\boldsymbol{d}}$ is the resultant vector. ${\boldsymbol{L}}$, the graph Laplacian, is defined as ${\boldsymbol{D}}{\boldsymbol{-}}{\boldsymbol{A}}$, where ${\boldsymbol{D}}$ is a diagonal matrix containing the degree of each node, and ${\bf{A}}$ is the graph adjacency matrix of the input network. The scalar parameter ${\boldsymbol{t}}$ represents the total diffusion time and controls the extent to which the original signal can spread across the network. We use a default value of *t* = 0.1. The term ***exp***($*$) denotes the matrix exponential. Given the sensitivity of the model’s performance to the network propagation parameter *t*, we conducted a sensitivity analysis (Supplementary Fig. S2). While *t* = 0.1 may not be the optimal choice, it is the default value commonly used in the Cytoscape heat diffusion plugin^72^, and we have adopted it.

### Methods for evaluating prognostic models

To evaluate the performance of prognostic models, several widely used methods are often combined. Here is a brief overview of each:

#### Concordance index

The Concordance index (C-index)^73^ measures the predictive accuracy of prognosis models by calculating the proportion of concordant pairs among all possible pairs in the study data. It is particularly useful for models predicting patient survival times or other time-related events. To compute the C-index, the input data included patient survival times or event occurrence times, observed status (with ‘1’ indicating an event occurrence and ‘0’ otherwise), and model-predicted scores for each patient. A pair is considered concordant if the patient with a longer actual survival has a higher predicted survival probability. Pairs, where neither patient experienced the event nor where one patient had not yet reached the event endpoint, were excluded from the calculation. The C-index is calculated as *K*/*M*, where *K* is the number of concordant pairs, and *M* is the total number of valid pairs evaluated.

#### Hazard ratio (HR)

The hazard ratio was used to assess and compare the accuracy of various prognostic methods. To facilitate personalized treatment planning by clinicians, patients are often divided into high-risk and low-risk groups based on the median of their predicted risk scores. These risk scores are binarized, and each patient is assigned to a respective risk group, R. A Cox proportional hazards model is then utilized to estimate the differences in risk between these two survival groups, as described below (3):

3 $${\rm{h}}({{\rm{t}}|{{\rm{R}}}})={{\rm{h}}}_{0}({\rm{t}}){\rm{exp}} ({{\upbeta}}{\rm{R}})$$

where ${h}_{0}(t)$ is the baseline hazard function. The term $\exp (\beta )$, defined as the hazard ratio (HR), quantifies the difference in risk between two patient groups. The HR quantifies the difference in risk, with a higher HR indicating a greater disparity in risk between the low-risk and high-risk groups, thus reflecting the superior performance of the predictive method.

#### KM survival curve

The Kaplan–Meier (KM) survival curves^74^ and the log-rank tests^75^ were used to determine whether the two risk groups had significantly different survival patterns. In a KM plot, the *Y*-axis shows the probability of survival over time, represented on the *X*-axis. Distinct, non-overlapping KM curves indicate clear differences in survival outcomes. The log-rank test was used to determine if the survival curves were statistically equivalent, with a *p*-value less than 0.05 indicating a significant difference, suggesting effective differentiation between groups.

### Improved stepwise algorithm for gene signature identification

Both node embedding and heat diffusion algorithms generate a ranked gene list based on either the similarity between gene expression and clinical phenotype variation or the heat value of each gene. Given that our training dataset comprises approximately 1500 samples, we selected the top 150 candidate genes, representing the top 10% of the ranked genes. To construct a robust prognostic model, we needed to filter a minority of genes from the ranked list. Traditional stepwise strategies—forward, backward, and both—do not adequately filter a moderate size for gene signatures. Consequently, we obtained 139, 43, and 41 genes, respectively, without a significant increase in the C-index (supplementary Table S7). Instead, we devised an improved greedy strategy to identify the gene signature.

Using the ranked gene list, we employed a multi-iteration approach to progressively reduce the number of genes until the size stabilized. In this process, if adding a new gene decreases the model’s performance (as measured by the C-index), we label that gene ‘hindering’. Conversely, if adding a gene increases the C-index, it is labeled ‘helpful’. Before the next iteration, we removed all ‘hindering’ genes and retained those labeled ‘helpful’. This operation is repeated in subsequent iterations until the size of the gene set no longer changes. The final set of genes forms the gene signature we require. The pseudocode outlining the algorithm for identifying gene signatures from a ranked gene list is provided (Supplementary Table S8). Iterations for gene signature identification for netHD are listed (Supplementary Fig. S3).

### Gene signature functional co-occurrence analysis

To assess the functional co-occurrence among breast cancer gene signatures within a specific cohort, we calculated the functional activity of each gene signature in each sample using the single-sample gene set enrichment analysis (ssGSEA) algorithm^53^. This process provided a set of functional activity scores for each gene signature across all patients. We then computed the Pearson correlation coefficient between these gene signatures. The resulting correlation values reflect the degree of functional co-occurrence among the signatures. To further analyze the clustering of gene signatures, a hierarchical clustering method was employed to extract cluster information.

## Supplementary information

Supplementary information

## Data Availability

All data utilized in this research are accessible publicly, with details provided in the Methods section. The paper includes web links and unique identifiers for the public cohorts and datasets used.

## Code availability

Source codes and data used to generate the results were deposited on GitHub https://github.com/immbal/NBT.
