# Supplementary material for: Network-based transfer of pan-cancer immunotherapy responses to guide breast cancer prognosis
Source: NPJ Syst Biol Appl. 2025 Jan 10;11:4. doi: 10.1038/s41540-024-00486-7 (PMC11720706; doi:10.1038/s41540-024-00486-7)
Supplement: Supplementary file 1 — Supplementary information [file 41540_2024_486_MOESM1_ESM.pdf]

Supplementary tables and figures for "Network-based transfer of pan-cancer immunotherapy responses to guide breast cancer prognosis"

Xiaobao Ding[1,2,3], Lin Zhang[1], Ming Fan[1*], Lihua Li[1,3*]

[1]Institute of Biomedical Engineering and Instrumentation, Hangzhou Dianzi University, 310018, Hangzhou, Zhejiang, China

[2]Institute of Big Data and Artificial Intelligence in Medicine, School of Electronics and Information Engineering, Taizhou University, 318000, Taizhou, Zhejiang, China

[3]School of Computer Science and Technology, Hangzhou Dianzi University,310018, Hangzhou, Zhejiang, China

Supplementary tables

Table S1: Lists of genes for the netHD and netNE methods

Gene signature	gene list
netHD	**GBP5** UBASH3A **GBP1** TRAT1 CD3E **IFNG** **IRF1** **LAG3** CD27 **CD8A** GBP2 CD8B **CXCL9** **CXCL10** TDO2 HLA-E FCGR1A GZMB HMGA1 CD70 CXCL12 CCR6 CCL25 CX3CL1 MAPK8IP2 TAPBP
netNE	GPR174 **GBP5** FCRL3 THEMIS **LAG3** **CXCL9** **GBP1** CXCL13 **CD8A** **CXCL10** HLA-G KLRC1 LILRA2 **IFNG** **IRF1** CXCL17 MS4A2 CD1C KIR3DL3 CCDC81 GRAP TNFRSF14

Table S2: Evaluation of the pan-cancer prognostic performance of netHD and netNE

TCGA_study	netType	OS*	DSS	PFI
BLCA	netHD	0.676	0.689	0.651
	netNE	0.653	0.7	0.642
HNSC	netHD	0.658	0.667	0.626
	netNE	0.663	0.647	0.585
KIRC	netHD	0.738	0.788	0.698
	netNE	0.724	0.73	0.645
LGG	netHD	0.81	0.811	0.717
	netNE	0.818	0.814	0.682
LIHC	netHD	0.667	0.668	0.654
	netNE	0.672	0.655	0.585
LUAD	netHD	0.667	0.664	0.573
	netNE	0.642	0.64	0.541
LUSC	netHD	0.588	0.624	0.568
	netNE	0.603	0.653	0.588
OV	netHD	0.608	0.609	0.566
	netNE	0.644	0.641	0.57
SKCM	netHD	0.65	0.652	0.566
	netNE	0.659	0.659	0.576
STAD	netHD	0.69	0.708	0.656
	netNE	0.657	0.659	0.615

* OS survival data were utilized for gene signature identification and prognostic model training, while DSS and PFI data were reserved for testing.

Table S3: All nine gene signatures used for benchmarking

Gene signature	Size	Function enrichment of signatures
Endo[1]	8	***Cell Cycle Checkpoints***; Mitotic Spindle Checkpoint; Separation of Sister Chromatids
GGI97[2]	97	***Cell Cycle Checkpoints***; Resolution of Sister Chromatid Cohesion; Mitotic Anaphase
LM[3]	54	Interleukin-4 and Interleukin-13 signaling; Extracellular matrix organization; Signaling by Interleukins
Mamma[4]	66	***Cell Cycle Checkpoints***; S Phase; G0 and Early G1
Pam50[5]	50	***Cell Cycle Checkpoints***; Mitotic Anaphase; Mitotic Metaphase and Anaphase
RS[6]	16	Transcriptional regulation by the AP-2 (TFAP2) family of transcription factors; TFAP2 (AP-2) family regulates transcription of growth factors and their receptors; Polo-like kinase mediated events
scP.W[7]	10	APC/C:Cdc20 mediated degradation of Cyclin B; Cdc20:Phospho-APC/C mediated degradation of Cyclin A; APC:Cdc20 mediated degradation of cell cycle proteins prior to satisfation of the ***cell cycle checkpoint***
netHD	26	***Interferon gamma signaling***; Chemokine receptors bind chemokines; ***Interferon Signaling***
netNE	22	***Interferon gamma signaling***; Immunoregulatory interactions between a Lymphoid and a non-Lymphoid cell; ***Interferon Signaling***

Table S4: Overview of the pan-cancer cohort for immune checkpoint therapy

Dataset	Tissue	R	NR	PD	SD	PR	CR
Choueiri2016	KIRC	3	13	8	5	3	0
Gide2019	SKCM	49	42	29	13	32	17
HugoLo2016	SKCM	14	13	13	0	10	4
IMVigor210	BLCA	68	230	167	63	43	25
IMmotion150	KIRC	48	117	52	65	32	16
Kim2018	STAD	12	33	18	15	9	3
Liu2019	SKCM	48	74	56	18	32	16
Miao2018	KIRC	5	12	8	4	4	1
Prins2019	GBM	0	28	19	9	0	0

Riaz2017	SKCM	20	78	44	34	14	6
VanAllen2015	SKCM	8	33	26	7	6	2

Table S5: Summary of bulk RNA-seq datasets for breast cancer

Dataset	Platform	Sample size	transcripts
TCGA	RNA-seq	987	20,513
METABRIC	microarray	1420	24,174
GPL9098	microarray	216	21,439

Table S6: Anchor genes associated with pan-cancer immunotherapy responses

CCL5	CD2	CD247	CD27	CD3E	CD3G	CD8A	CXCL10
CXCL13	CXCL9	CXCR2P1	EOMES	FASLG	FCRL3	GBP1	GBP4
GBP5	GPR174	GZMH	HLA-DMA	HLA-F	ID01	IFNG	IL2RB
IRF1	ITGB7	KLRK1	LAG3	LCK	MAP4K1	PLA2G2D	PRF1
PYHINI	SLAMF7	TBX21	THEMIS	TIGIT	TRAT1	UBASH3A	

Table S7: Evaluation of stepwise strategies for parameter selection in Cox regression

Stepwise strategy	Concordance index	Final size after stepwise
forward	0.605	139
backward	0.656	41
both	0.657	43
Our	0.651	26

Table S8: Pseudocode for a custom algorithm for gene signature identification

Algorithm generateGeneSignature

Input: rankedGeneList: List of ranked gene list.
Output: selectedGenes: Optimized list of genes for gene signature.

```
procedure GENERATEGENESIGNATURE(rankedGeneList)
    candidateGenes ← initialGeneList
    convergent ← False
    ciPrev ← 0    # Initialize the previous CI to zero
    while not convergent
```

```
        selectedGenes ← empty list
        for n in 1 to length(candidateGenes)
            newCI ← CALCULATECI(candidateGenes[1:n])
            if newCI > ciPrev
                append candidateGenes[n] to selectedGenes
            ciPrev ← newCI
        if length(selectedGenes) == length(candidateGenes)
            convergent ← True
        else
            candidateGenes ← selectedGenes
    return selectedGenes
end procedure
function CALCULATECI(geneList)
    # Implement CI calculation here
    return CI_value
end procedure
```

Supplementary figures

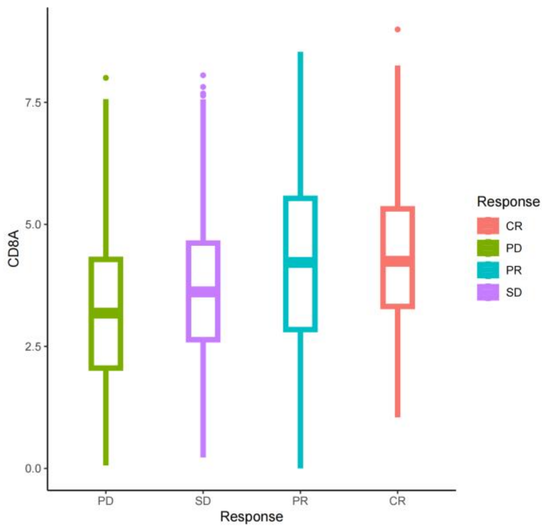

Figure S1: CD8A expression levels across immunotherapy response groups. Box plot illustrating CD8A expression levels in a pan-cancer cohort, stratified by immunotherapy response groups: progressive disease (PD), stable disease (SD), partial response (PR), and complete response (CR). Colors indicate response types: green for PD, purple for SD, blue for PR, and red for CR.

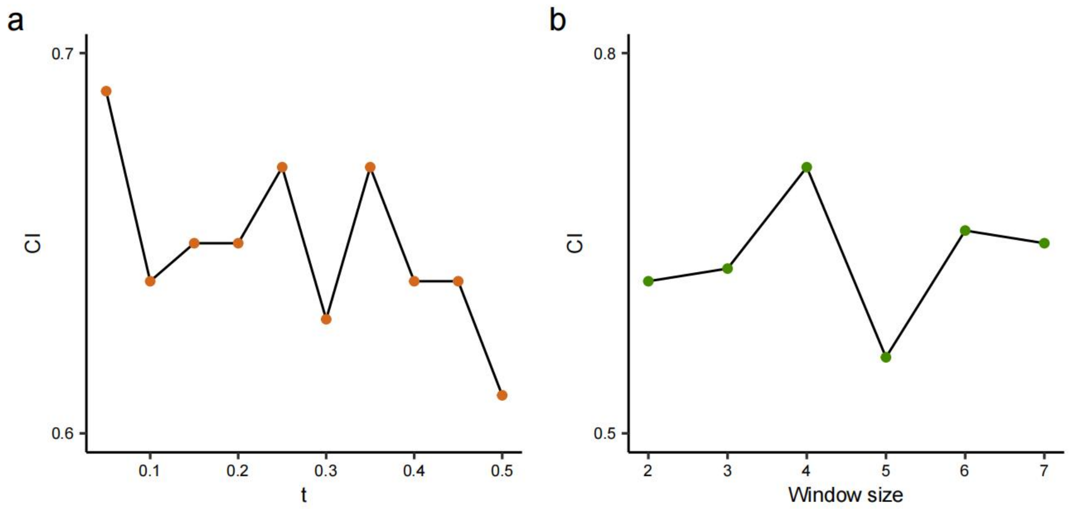

Figure S2: Parameter sensitivity analysis for heat diffusion and network embedding. (a) Sensitivity analysis of the heat diffusion parameter t, ranging from 0.05 to 0.5 in steps of 0.05.

The y-axis represents the concordance index for model performance. (b) Sensitivity analysis of the network embedding parameter (window size), ranging from 2 to 7 in steps of 1. The y-axis represents the concordance index for model performance.

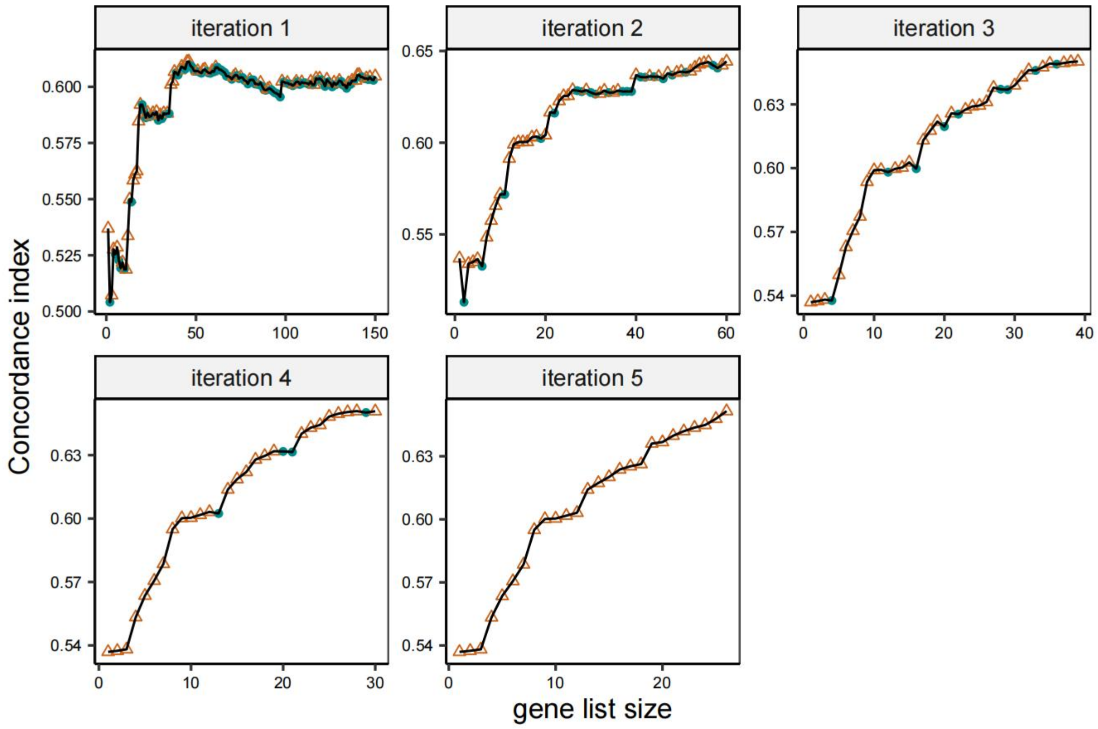

Figure S3: Model performance over iterations for the netHD-ranked gene list. The x-axis shows the size of the added gene list, while the y-axis indicates the concordance index for the respective gene list. Each facet represents a different iteration. Upward-facing triangles denote 'helpful' genes, and circles represent 'hindering' genes.

References

[1] M. Filipits, M. Rudas, R. Jakesz, P. Dubsky, F. Fitzal, C.F. Singer, O. Dietze, R. Greil, A. Jelen, P. Sevelda, C. Freibauer, V. Muller, F. Janicke, M. Schmidt, H. Kolbl, A. Rody, M. Kaufmann, W. Schroth, H. Brauch, M. Schwab, P. Fritz, K.E. Weber, I.S. Feder, G. Hennig, R. Kronenwett, M. Gehrmann, M. Gnant, E.P. Investigators, A new molecular predictor of distant recurrence in ER-positive, HER2-negative breast cancer adds independent information to conventional clinical risk factors, Clin Cancer Res 17(18) (2011) 6012-20.

[2] C. Sotiriou, P. Wirapati, S. Loi, A. Harris, S. Fox, J. Smeds, H. Nordgren, P. Farmer, V. Praz, B. Haibe-Kains, C. Desmedt, D. Larsimont, F. Cardoso, H. Peterse, D. Nuyten, M. Buyse, M.J. Van de Vijver, J. Bergh, M. Piccart, M. Delorenzi, Gene expression profiling in breast cancer: understanding the molecular basis of histologic grade to improve prognosis, J Natl Cancer Inst 98(4) (2006) 262-72.

[3] A.J. Minn, G.P. Gupta, P.M. Siegel, P.D. Bos, W. Shu, D.D. Giri, A. Viale, A.B. Olshen, W.L. Gerald, J. Massague, Genes that mediate breast cancer metastasis to lung, Nature 436(7050) (2005) 518-24.

[4] L.J. van 't Veer, H. Dai, M.J. van de Vijver, Y.D. He, A.A. Hart, M. Mao, H.L. Peterse, K. van der Kooy, M.J. Marton, A.T. Witteveen, G.J. Schreiber, R.M. Kerkhoven, C. Roberts, P.S. Linsley, R. Bernards, S.H. Friend, Gene expression profiling predicts clinical outcome of breast cancer, Nature 415(6871) (2002) 530-6.

[5] J.S. Parker, M. Mullins, M.C. Cheang, S. Leung, D. Voduc, T. Vickery, S. Davies, C. Fauron, X. He, Z. Hu, J.F. Quackenbush, I.J. Stijleman, J. Palazzo, J.S. Marron, A.B. Nobel, E. Mardis, T.O. Nielsen, M.J. Ellis, C.M. Perou, P.S. Bernard, Supervised risk predictor of breast cancer based on intrinsic subtypes, J Clin Oncol 27(8) (2009) 1160-7.

[6] S. Paik, S. Shak, G. Tang, C. Kim, J. Baker, M. Cronin, F.L. Baehner, M.G. Walker, D. Watson, T. Park, W. Hiller, E.R. Fisher, D.L. Wickerham, J. Bryant, N. Wolmark, A multigene assay to predict recurrence of tamoxifen-treated, node-negative breast cancer, New England Journal of Medicine 351(27) (2004) 2817-2826.

[7] X. Li, L. Liu, G.J. Goodall, A. Schreiber, T. Xu, J. Li, T.D. Le, A novel single-cell based method for breast cancer prognosis, PLoS Comput Biol 16(8) (2020) e1008133.
